# Low oxygen levels as a trigger for enhancement of respiratory metabolism in *Saccharomyces cerevisiae*

**DOI:** 10.1186/1471-2164-10-461

**Published:** 2009-10-05

**Authors:** Eija Rintala, Mervi Toivari, Juha-Pekka Pitkänen, Marilyn G Wiebe, Laura Ruohonen, Merja Penttilä

**Affiliations:** 1 VTT Technical Research Centre of Finland, P.O. Box 1000, FI-02044 VTT, Finland

## Abstract

**Background:**

The industrially important yeast *Saccharomyces cerevisiae *is able to grow both in the presence and absence of oxygen. However, the regulation of its metabolism in conditions of intermediate oxygen availability is not well characterised. We assessed the effect of oxygen provision on the transcriptome and proteome of *S. cerevisiae *in glucose-limited chemostat cultivations in anaerobic and aerobic conditions, and with three intermediate (0.5, 1.0 and 2.8% oxygen) levels of oxygen in the feed gas.

**Results:**

The main differences in the transcriptome were observed in the comparison of fully aerobic, intermediate oxygen and anaerobic conditions, while the transcriptome was generally unchanged in conditions receiving different intermediate levels (0.5, 1.0 or 2.8% O_2_) of oxygen in the feed gas. Comparison of the transcriptome and proteome data suggested post-transcriptional regulation was important, especially in 0.5% oxygen. In the conditions of intermediate oxygen, the genes encoding enzymes of the respiratory pathway were more highly expressed than in either aerobic or anaerobic conditions. A similar trend was also seen in the proteome and in enzyme activities of the TCA cycle. Further, genes encoding proteins of the mitochondrial translation machinery were present at higher levels in all oxygen-limited and anaerobic conditions, compared to fully aerobic conditions.

**Conclusion:**

Global upregulation of genes encoding components of the respiratory pathway under conditions of intermediate oxygen suggested a regulatory mechanism to control these genes as a response to the need of more efficient energy production. Further, cells grown in three different intermediate oxygen levels were highly similar at the level of transcription, while they differed at the proteome level, suggesting post-transcriptional mechanisms leading to distinct physiological modes of respiro-fermentative metabolism.

## Background

Oxygen is one of the basic determinants of cellular physiology. Oxygen is needed for energy metabolism and sterol, fatty acid and heme biosynthesis, but may also cause oxidative damage, especially when cells are exposed to oxygen after being in oxygen-restricted conditions [1]. Regulation of metabolism in response to oxygen availability is needed for rapid adaptation to changing environments both in nature and in industrial bioprocesses. *Saccharomyces cerevisiae*, a major industrial organism, is able to grow both in the presence and in the complete absence of oxygen by adjusting the mode of metabolism from respiratory to respirofermentative and fermentative. Among yeasts, *S. cerevisiae *and other *Saccharomyces *species are unique in being able to restrict respiration and increase fermentative metabolism on glucose, even in the presence of oxygen, by the repression of respiratory genes [2].

The concentration of heme plays a central role in the regulation of oxygen responsive genes in *S. cerevisiae*, through the biosynthetic pathway of heme which is not active in the absence of oxygen. However, there are at least two types of heme pools in the cell, a protein-bound and a free pool, and it is not known how these two pools contribute to the transcriptional regulation [3]. The transcription factor Hap1p acts as an activator or as a repressor of certain genes depending on the presence or absence of heme. In the presence of heme, Hap1p activates the expression of genes involved in respiration and oxidative stress [4,5]. Transcriptional activation by Hap1p increases dramatically between anaerobic and severely oxygen- restricted conditions, but only gradually between 1 μM O_2 _and fully aerobic conditions [3]. Hap1p also induces the expression of *ROX1*, which encodes a repressor of genes needed during severe hypoxia or in anaerobic conditions [6,7]. In the absence of heme, Hap1p acts as a repressor of genes involved in ergosterol biosynthesis [8]. The transcription factor Hap2/3/4/5p is also suggested to be activated by heme and it induces the expression of many genes involved in respiratory metabolism in the presence of oxygen [9,10]. However, while the regulation of Hap1p by heme has been widely studied, the regulation of Hap2/3/4/5p by heme and oxygen is largely unknown [11].

In anaerobiosis, the cell wall and cell membrane of *S. cerevisiae *is remodelled, which enables import of sterols and fatty acids, which, like heme, are not synthesised in the absence of oxygen [9,12-16]. Transcription factors Upc2p, Ecm22p and Sut1p are known to play a role in the import of sterols, but the exact mechanisms are not known [17,18]. However, nearly one third of anaerobically upregulated genes contain Upc2p/Ecm22p binding sites in their promoters [19,20]. Upc2p and Ecm22p bind the same sequence and the binding is dependent on sterol concentration [21]. In addition, Mox1p and Mox2p have been suggested to be repressors interacting with Upc2p [22]. The target genes of Sut1p are not known, but the overexpression of *SUT1 *has been shown to enable uptake of sterols in aerobic conditions [23,24].

Genome wide studies have revealed that a large part of the *S. cerevisiae *transcriptome reacts to the presence or absence of oxygen, partly depending on the carbon source and nutrient limitation [12-14,25,26]. While Piper and co-workers identified 877 transcripts differentially expressed between aerobic and anaerobic glucose-limited conditions, Tai and co-workers found that only 155 of these genes responded consistently to anaerobiosis under four different macronutrient limitations [25,26]. Lai and co-workers monitored the transcriptome of *S. cerevisiae *during the transition from aerobic to anaerobic conditions in batch cultivations on glucose and galactose [13,14]. These studies revealed an initial response of stress-activated genes only on galactose, while later responses of downregulation of mitochondrial functions, upregulation of carbohydrate metabolism and redox regulation and activation of networks involved in sterol and cell wall homeostasis were similar on both carbon sources. In addition to transcriptome analyses, a recent comparison of the transcriptome and proteome revealed post-transcriptional regulation of glycolysis and of the aminoacyl-tRNA, purine and amino acid biosynthetic pathways, in respect to oxygen availability [27].

To our knowledge, there is no published data of the transcriptome or proteome in steady state conditions with intermediate oxygen levels. Studies of yeast provided with different oxygen levels could reveal regulation that is dependent not only on the presence or absence oxygen, but also on oxygen concentration. Severe hypoxia is known to modulate gene expression of some gene pairs in a Hap1p, Hap2/3/4/5p and Rox1p dependent manner and it is thought to enable more efficient oxygen utilisation. *COX5a*/*COX5b*, *CYC1/CYC7, AAC2/AAC3 *and *TIF51a/ANB1 *are pairs of interchangeable genes, of which one member of the pair is used under aerobic conditions and the other under severe oxygen restriction [9]. This switch occurs only in very low oxygen concentrations [28] and nothing is known about the expression of these gene pairs under conditions of moderately low oxygen.

Under steady state glucose-limited conditions, glucose repression of respiratory functions does not occur and it is possible to study the effect of oxygen on metabolism without interfering effects of using different carbon sources or changes in the specific growth rate. We cultivated *S. cerevisiae *in highly controlled glucose-limited chemostat cultures with 0, 0.5, 1.0, 2.8 and 20.9% oxygen in the feed gas and studied the levels of selected transcripts, metabolites and fluxes of central carbon metabolism [29,30]. Our studies revealed that cells grown with 2.8% oxygen in the feed gas were very similar to those grown with 20.9% oxygen (fully aerobic conditions) in terms of oxygen uptake rate, carbon evolution rate, and biomass production, while only minor changes in fluxes were seen. However, the metabolism was already respiro-fermentative with 2.8% oxygen and a large fraction of measured transcripts levels differed from those observed in cells grown with 20.9% oxygen [29,30]. Furthermore, even though the biomass yield and the respirative carbon flux through the TCA cycle were significantly reduced when cells were fed 1.0% or 0.5% oxygen, compared to fully aerobic conditions, 36% and 25% of the ATP was still generated through respiration with 1.0% and 0.5% oxygen, respectively [29,30]. In order to get a global view on the metabolism of *S. cerevisiae *under various conditions of oxygen provision, we have performed whole transcriptome and partial proteome analysis of *S. cerevisiae *cells grown in glucose-limited chemostat cultures with 0, 0.5, 1.0, 2.8 or 20.9% oxygen in the feed gas and used both well established and recently published computational tools for a thorough analysis of the data.

## Results

### The effect of oxygen provision on gene transcription in steady state glucose-limited chemostats

**Figure 1:**
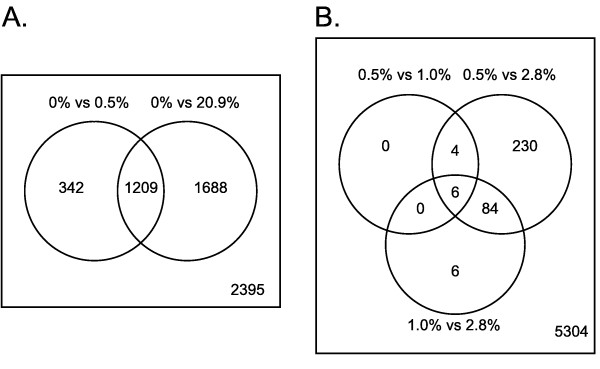
**Venn diagrams of the genes which differ significantly (p < 0.01) in conditions of different oxygen provision in the feed gas**. A. Anaerobic and either 0.5 or 20.9% oxygen in the feed gas and B. 0.5% and 1.0%, 0.5% and 2.8%, and 1.0% and 2.8% oxygen in the feed gas. The number in the lower right corner of the figures A and B represents the number of genes that were not differentially expressed.

Microarray analysis of yeast from glucose-limited chemostat cultivations with 0, 0.5, 1.0, 2.8 and 20.9% oxygen in the feed gas was performed. Statistical analysis of the steady state data revealed that 3435 genes responded significantly (p < 0.01) to oxygen availability under the five conditions studied. While the highest number of responsive genes (2900) was observed between the anaerobic and fully aerobic conditions, the number of genes expressed differently in conditions of intermediate oxygen (0.5-2.8%) was relatively small (Figure 1A and 1B). The transcriptome from cultures with 0.5% and 1.0% oxygen was particularly similar: only 10 genes had statistical differences (p < 0.01) in their expression. When the anaerobic or fully aerobic conditions were compared to conditions of intermediate oxygen, significant differences were found in 2000-2400 and 1500-1600 genes, respectively.

To obtain an overall picture of metabolic pathways responding to oxygen availability, gene set enrichment analysis was performed. This analysis allows the identification of defined sets of genes with differential expression between two classes of samples [31,32]. Parametric gene set enrichment analysis (PAGE) uses fold changes between experimental groups to calculate Z scores for predefined gene sets and uses normal distribution to infer the statistical significance of the gene sets [33]. This approach was used in the present study to identify KEGG pathways and GO categories (containing 10 or more genes) which contained genes that were differentially expressed in conditions of different oxygen provision. Pair wise comparisons of successive oxygen levels and of the anaerobic and fully aerobic conditions are shown in Table 1. Comparison of intermediate oxygen levels showed that few pathways were differentially expressed when cells were provided with 0.5, 1.0 or 2.8% oxygen. In particular, comparison of 0.5% and 1.0% oxygen found no statistically significant differences, even at a p-value of 0.05 (data not shown).

**Table 1:** Parametric gene set enrichment analysis of GO classes and KEGG pathways

**GO category**	**p-value**	**KEGG pathway**	**p-value**
**0% vs. 0.5 or 1.0%**		**0% vs. 0.5 or 1.0%**	
Sterol transport	2.83E-08	Oxidative phosphorylation	6.63E-12
Pheromone-dependent signal transduction during conjugation with cellular fusion	5.20E-06	Arginine and proline metabolism	1.59E-09
Aerobic respiration	0.0009	Glutathione metabolism	1.26E-05
Glutamate biosynthetic process	0.0015	Citrate cycle (TCA cycle)	1.74E-05
Tricarboxylic acid cycle	0.0025	MAPK signalling pathway	2.87E-05
Exocytosis	0.0065		
			

**0% vs. 2.8%**		**0% vs. 2.8%**	
Sterol transport	1.67E-07	Arginine and proline metabolism	2.50E-13
Pheromone-dependent signal transduction during conjugation with cellular fusion	8.38E-06	Citrate cycle (TCA cycle)	4.65E-07
Glutamate biosynthetic process	7.23E-05	MAPK signalling pathway	0.0002
Tricarboxylic acid cycle	0.0006	Porphyrin metabolism	0.0009
Aerobic respiration	0.0032	Sulphur metabolism	0.0039
Exocytosis	0.0056		
			

**0% vs. 20.9%**		**0% vs. 20.9%**	
Response to stress	1.54E-06	Glyoxylate and dicarboxylate metabolism	2.77E-17
Sterol transport	2.27E-05	Citrate cycle (TCA cycle)	3.16E-12
Meiosis	0.0008	Bile acid biosynthesis	3.05E-08
NADH oxidation	0.0025	Ascorbate and aldarate metabolism	5.83E-07
Sporulation	0.0063	Nucleotide sugars metabolism	3.18E-05
Sphingolipid biosynthetic process	0.0075	Propanoate metabolism	7.84E-05
		Fatty acid metabolism	0.0002
		Pentose phosphate pathway	0.0004

**0.5% vs. 1.0%**		**0.5% vs. 1.0%**	

**0.5 or 1.0% vs. 2.8%**		**0.5 or 1.0% vs. 2.8%**	
		Glyoxylate and dicarboxylate metabolism	6.15E-20
		Fatty acid metabolism	0.0001
		Porphyrin metabolism	0.0002

**0.5 or 1.0% vs. 20.9%**		**0.5 or 1.0% vs. 20.9%**	
Sporulation (sensu Fungi)	0.0015		
Pheromone-dependent signal transduction during conjugation with cellular fusion	0.0027		
Meiosis	0.0048		

**2.8% vs. 20.9%**		**2.8% vs. 20.9%**	
Response to stress	1.1E-12	Glyoxylate and dicarboxylate metabolism	3.93E-06
Sporulation (sensu Fungi)	0.0003	Phenylalanine metabolism	0.0064
Protein folding	0.0013	Amino sugars metabolism	
Pheromone-dependent signal transduction during conjugation with cellular fusion	0.0017		
SRP-dependent co-translational protein targeting to membrane, translocation	0.0019		
Hexose transport	0.0032		
Iron ion homeostasis	0.0077		

Pair wise comparison of transcriptome data from cells grown in glucose-limited chemostats receiving 0, 0.5, 1.0, 2.8 or 20.9% oxygen (p-values < 0.01). For 0.5 and 1.0% oxygen, the data has been combined and the p-values are averaged p-values.

Most of the genes (78%) which were differentially expressed between anaerobic and 0.5% provided oxygen were likewise differentially expressed between anaerobic and fully aerobic conditions (Figure 1A). PAGE analysis revealed that the pathways that were differentially expressed between anaerobic and 0.5 or 1.0% provided oxygen, but not between anaerobic and fully aerobic conditions were those of oxidative phosphorylation, pheromone signalling, arginine, proline and glutathione metabolism and exocytosis (Table 1). Pathways unique to the comparison of 2.8% and 20.9% provided oxygen were protein folding, iron ion homeostasis, protein targeting to membrane and metabolism of phenylamine and amino sugars.

### Clustering of transcription data and promoter analysis of the clusters

**Figure 2:**
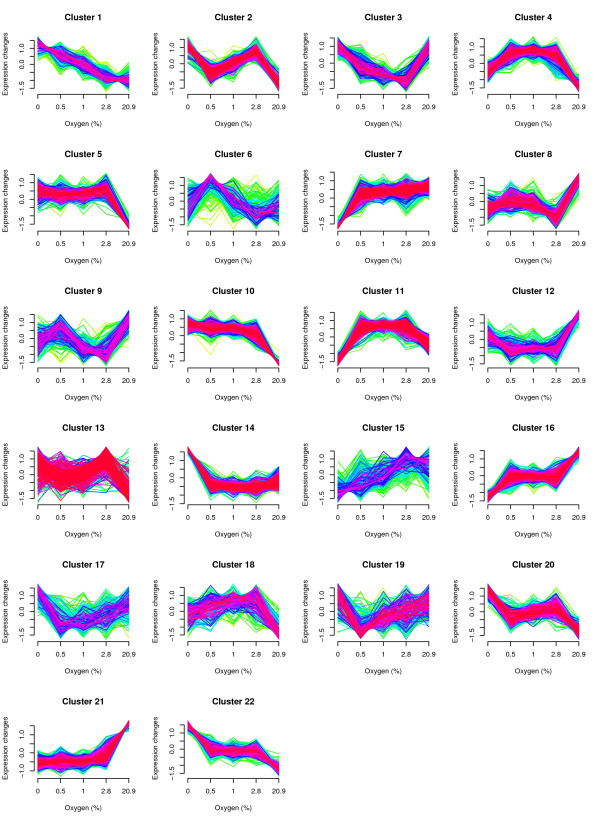
**Fuzzy c-means clustering of gene expression patterns in cells grown with 0, 0.5, 1.0, 2.8 and 20.9% oxygen in the feed gas**. The clustering was performed with individual samples, but average values for each condition are shown in the graphs. The expression values are centred and scaled around a mean of zero and standard deviation of 1, for all the genes. Red and purple represent genes that have membership values higher than 0.5 while green and yellow represent genes that have membership values below 0.5.

Cluster analysis of the transcriptional data was carried out using fuzzy c-means clustering, which enabled clustering without prefiltering of the genes and thus included potentially interesting genes that did not differ strongly in the different conditions and which would otherwise have been discarded from the analysis [34]. Fuzzy c-means clustering is a soft clustering method that assigns genes to clusters with gradual membership values between zero and one. Not all genes are forced into clusters, as is often the case in traditional clustering of predetermined, significantly changing genes. Moreover, the membership values for the clusters can be used to determine the level of coregulation under consideration. The fuzzy c-means clustering of gene expression data from *S. cerevisiae *cultures grown with different amounts of oxygen and the most significant over-represented GO-categories and KEGG-pathways in these clusters are presented in Figure 2 and Additional file 1), respectively. Analysis of the gene expression data revealed 22 clusters containing 37-267 genes with alpha values higher than 0.5, i.e. the genes belonged with highest probability to the respective cluster.

**Figure 3:**
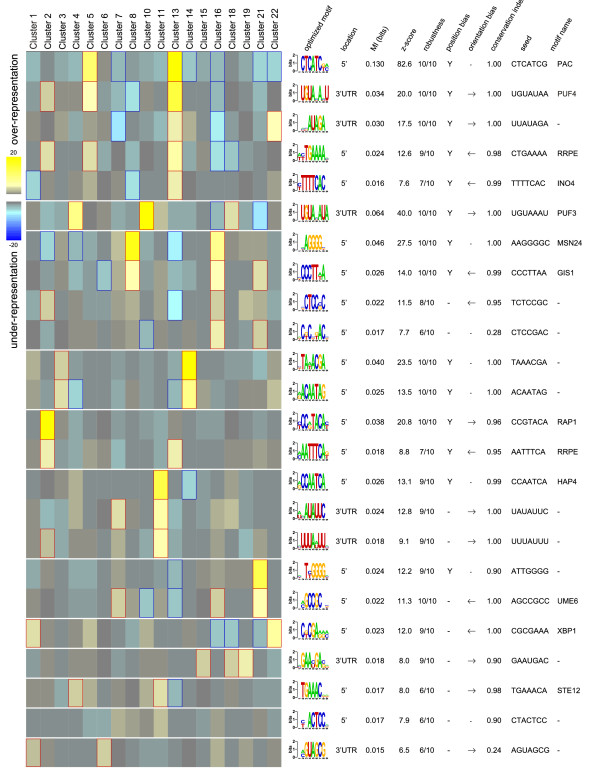
**FIRE analysis for transcriptional regulatory motifs occurring in the clusters presented in figure 2**. For each cluster, the most significant GO enrichments are shown at the top. Yellow indicates over-representation of a motif in a given cluster and significant (p < 0.05) overrepresentation is highlighted with red frames. Similarly, blue blocks and blue frames indicate significant (p < 0.05) under-representation. For each motif, the location (either 5' upstream or 3' UTR), mutual information (MI) value, Z score associated with the MI value, a robustness score ranging from 1/10 to 10/10, a position bias indicator ("Y" indicates position bias is observed), orientation bias indicator, conservation index, the seed that gave rise to the motif and name of the closest known motif are presented. For more details, see Elemento and co-workers 2007 [35].

The promoter and 3'UTR sequences of genes in the clusters identified using fuzzy c-means clustering were analysed using FIRE software [35] and the results of the analysis are shown in Figure 3. The analysis revealed 17 transcription factor binding site motifs and 7 3'UTR motifs, of which some had significant co-occurrence and/or co-localisation patterns. A more detailed description of the results of clustering and promoter analysis is provided below.

### Genes of the respiratory pathway and TCA-cycle have enhanced expression in intermediate compared to fully aerobic conditions

Two steady state clusters (cluster 4 and cluster 11) contained genes that had higher expression in all intermediate oxygen conditions compared to either anaerobic or fully aerobic conditions. The transcription levels of genes in cluster 4 were higher in anaerobic than aerobic conditions, while the opposite was observed in cluster 11. Cluster 4 was enriched in genes of KEGG pathways for the cell cycle and glycerophospholipid metabolism, while cluster 11 was enriched in genes related to oxidative phosphorylation, the TCA cycle, the MAPK signalling pathway and pyruvate metabolism. FIRE analysis revealed that different motifs were enriched in the promoters and 3'UTR sequences of the genes of these two clusters. In genes of cluster 4, motifs for Puf3p 3'UTR sites were found, while genes in cluster 11 were enriched in binding sites of the Hap2/3/4/5p transcription factor and two previously undescribed 3'UTR motifs (WHATATTC and HTTTAWTTH). All three motifs found in cluster 11 had significant co-occurrence amongst the genes.

Nearly all of the genes encoding nuclear-encoded subunits of respiratory chain complexes were located in cluster 11 (30 out of 37) and cluster 4 (4 out of 37), thus having their highest expression levels in the intermediate oxygen conditions. Cluster 11 and 4 also contained genes encoding several TCA cycle enzymes: Cit1p, Aco1p, Idh1p, Kgd1p, Kgd2p, Lpd1p, Mdh1p (cluster 11) and Idh2p (cluster 4). The increase in the expression was mainly less than 2-fold, suggesting a subtle change of the components of these pathways. Of the genes encoding the main enzymes of the TCA cycle, only *FUM1, LSC1 *and *LSC2 *did not have their highest expression level in the intermediate oxygen conditions, but in the fully aerobic conditions. Further, genes encoding isoenzymes of the enzymes of the TCA cycle had their highest expression either in fully aerobic (*IDP2, IDP3, MDH2*, *MDH3, CIT3, YLR164W, YJL045W*, *YMR118C*) or anaerobic (*CIT2*) conditions.

Many respiratory enzymes contain metals and accordingly, many genes involved in metal transport and homeostasis were found in clusters 4 and 11. Genes encoding vacuolar iron transporters Fth1p and Fet5p, plasma membrane copper transporters Ccc2p and Ctr1p, the metal ion transporter Smf1p and iron and copper reductase Fre1p were found in cluster 11. Additionally, genes encoding metallopeptidases/proteases Yta12p, Axl1p, Qri7p, and the copper deprivation induced ORF *YOR296W *were amongst the members of this cluster. Cluster 4 contained genes encoding plasma membrane siderophore-iron transporter Arn1p, oxidoreductase Fet3p, vacuolar zinc transporter Zrc1p and Ggc1p involved in mitochondrial iron homeostasis. Comparing gene expression in 2.8% oxygen and the fully aerobic conditions, 9 out of 16 genes known to be involved in transport of iron from the extracellular medium to the cytosol [36] had 2-16 fold higher expression and only two genes had lower expression in 2.8% oxygen than in the fully aerobic conditions.

Cluster 4 was enriched in genes related to mitochondrial organisation and biogenesis (*RPM2, POR1, UTH1, PNT1, CLU1, DNM1, MGM1, MBA1*). In addition, genes encoding mitochondrial translation elongation factors (*TUF1, MEF1*), mitochondrial translational activators (*CBS2, PET309*), mitochondrial ribosome recycling factor (*RRF1*) and subunits of mitochondrial ribosomes (10 genes) were found in this cluster. Cluster 10, in which the lowest level of expression occurred in the fully aerobic conditions and similar, higher expression levels occurred in the oxygen-limited and anaerobic conditions, also contained genes related to mitochondrial protein synthesis. 57 genes encoding components of mitochondrial ribosomes and 10 genes of mitochondrial protein import machinery were found in cluster 10. The 3' UTR motif for binding of Puf3p, which promotes degradation of mRNAs of nuclear-encoded mitochondrial proteins, was over-represented both in clusters 4 and 10. The expression of *PUF3 *itself was low and remained constant under all the conditions of different oxygen provision studied.

### Effect of oxygen on transcription of genes involved in lipid metabolism

Clusters 16 and 21 were enriched in genes related to fatty acid oxidation and peroxisomal biogenesis. Cluster 16 showed highest expression in fully aerobic conditions, lowest expression in anaerobic conditions and a similar, intermediate level of expression in all the intermediate oxygen conditions. Genes encoding activities of fatty acid β-oxidation (*TES1, POX1, CTA1, PXA1, SPS19, DCI1, ANT1, FOX2, POT1, PEX11, PXA2*), the oleate responding transcription factor *OAF1 *and 4 genes related to peroxisomal biogenesis (*PEX15, PEX2, PEX8, PEX18*) were located in this cluster. Gene expression in cluster 21 was at its highest in fully aerobic conditions, and at a lower, comparable level in the oxygen-limited and anaerobic conditions. This cluster contained 6 genes (*PCD1, YOR084W, CAT2, IDP3, ECI1, AAT2*) related to fatty acid metabolism, and 7 genes related to peroxisomal biogenesis (*PEX14*, *PEX5, PEX19, PEX30, PEX28, PEX1, PEX3, YMR018W*). The oleate responding transcription factor *PIP2 *was also located in this cluster.

Clusters 3 and 14 were enriched in genes related to sterol metabolism. Genes of cluster 3 were transcribed at lower levels in intermediate oxygen conditions, compared to fully aerobic or anaerobic conditions. The cluster contained genes encoding activities of ergosterol biosynthesis (*ERG6, ERG11, HMG2, ERG25, DAP1*), sterol transport (*SUT2, OSH2*), sterol homeostasis (*TGL1*) and synthesis of membrane sterols (*ATG26*). Genes in cluster 14 were transcribed at a lower level in all oxygen containing conditions, compared to anaerobic conditions. The cluster was enriched in genes encoding proteins involved in ergosterol biosynthesis (*ERG26, ERG7, ERG2, ERG3, ERG1, ERG10, NCP1, ERG9, ERG27, ERG24, ERG28, HES1*), sterol esterification (*ARE1*), sterol transport (*AUS1, SWH1*) and regulation of sterol transport and biosynthesis (*UPC2, ECM22*). Also *DAN/TIR *genes, encoding cell wall mannoproteins, and *PAU *genes of unknown function were accumulated in cluster 14 (*DAN1-4, TIR1-4, PAU2,3,5,9*). When a less strict α-value of 0.1 was used to define the genes belonging to this cluster, three additional *PAU *genes were found in it (*PAU7,17,18*).

Promoters of the genes in clusters 3 and 14 were enriched in two putative transcription factor binding sites that had strong, positive co-occurrence. The motif BTAWACGA was found in all the sterol metabolism-related genes of cluster 14, except in *SWH1*, and in all the three *ERG *genes of cluster 3. The motif RACAATAG was found in the promoters of 11 out of the 29 genes related to sterol metabolism of cluster 14, and in 2 out of 9 of those in cluster 3.

### Oxygen dependent stress responses

Three clusters (clusters 3, 8 and 16), with distinct expression profiles, showed enrichment in genes in the GO category of stress response, and binding sites of stress-related transcription factors Msn2/4p and Gis1p were over-represented among the promoters of the genes in two of these clusters (clusters 8 and 16). In the promoters of the genes in cluster 16, binding sites of Ume6p and two unknown transcription factors were also over-represented while, binding sites for a stress-activated transcriptional repressor Xbp1p were under-represented. Further, the gene encoding Xbp1p was a member of cluster 16. The expression level of *XBP1 *was induced 3-fold in the intermediate oxygen (0.5-2.8%) and 8-fold in the fully aerobic conditions compared to the anaerobic conditions. Promoter analysis revealed enrichment of the binding site for Xbp1p in clusters 1 and 22. These clusters had an average correlation of -0.81 and -0.97, respectively, to the expression level of *XBP1*. 72% and 68% of the genes in clusters 1 and 22, respectively, contained the central core bases (CTCGA) of the Xbp1p binding site. Many of these genes are related to the regulation of cell division (*GIC1, BUD4, TOS4, KIP2, TOS1, KIN4, TUB4, CIN8, TUB3, VIK1, SMC2, UNG1, PIN4, FKH1*) and cell wall organisation (*EXG2*, ORF YFL052W, *TOS1, BUD7, MHP1, DSE1, SUN4*).

### The MAPK signalling pathway for pheromone response and filamentous growth is affected by oxygen availability

Clusters 4, 7 and 11, of which clusters 4 and 11 have been discussed above with reference to genes involved in the TCA cycle and respiration, and which contain those genes which were more highly expressed in the conditions of intermediate oxygen availability, were enriched in genes involved in mating and filamentous growth. These clusters contained genes which showed a low level of expression in anaerobic, compared to intermediate oxygen conditions. However, they differed in the fully aerobic conditions, genes of clusters 4 and 11 had lower expression levels in the aerobic than in the intermediate oxygen conditions, but in cluster 7 the expression levels were comparable in all conditions provided with oxygen.

Genes in cluster 11 included some encoding proteins of the MAPK signalling pathways for pheromone response and filamentous growth (Ste3p, Gpa1p, Fus3p, Sst2p, Kss1p), genes regulated by these signalling pathways (*FUS2, FUS1, FIG1, SAG1, FIG2, PRM6, AGA1, PRM1, CLN1, BUD8, MSB2, CWP1, GFA1, KTR2, SVS1*) and the transcription factors (Ste12p, Tec1p) that are activated by these pathways. According to FIRE analysis, this cluster as well as cluster 4, which contained a set of genes related to mating (*FAR1, STE4, CLN2, MSG5, STE23, KAR5, ASH1, HO, CCW12*), were enriched in genes whose promoters contain the transcription factor binding site for Ste12p. Cluster 7 contained genes regulated by the MAPK signalling pathway for mating (*PRM5, PRM10, AGA2, MDG1, AFR1, PRR2, PRM8, CHS1*). While promoters of genes in cluster 7 were overall enriched with a binding site of Ume6p transcription factor, Ume6p binding site was not enriched in the promoters of the genes related to pheromone signalling.

### Comparison with previous data and oxygen dependence of genes of pentose phosphate pathway

We previously published transcription data for 72 selected genes related to central carbon metabolism, measured with the TRAC method [29]. Of those genes analysed with both Affymetrix (p < 0.01) and with TRAC (p < 0.05) methods, 61 showed statistically significant differences in their expression levels with both methods. Sixteen of the significantly changing genes showed >3-fold difference in expression and had an average correlation of 0.8 between the TRAC and the Affymetrix analysis. Thirteen of the significantly changing genes showed 2 to 3-fold difference in expression and had an average correlation of 0.6. Twenty-four of the significantly changing genes had <2-fold difference in their expression and had an average correlation of only 0.2. However, five of these genes which had <2-fold difference had correlations > 0.7. The genes that showed poor correlation between the TRAC and the Affymetrix data, and that showed ≥ 2-fold differences in the Affymetrix were *GPD2, CIT2, ACS1*, *HAP1, MAE1 *and *PCK1*, the signals of the three latter genes being very close to the detection limit using the TRAC method.

Large changes in the expression of *SOL4, GND2, TKL2 *and the ORF *YGR043C*, from the pentose phosphate pathway, were observed in Affymetrix data. These genes had their highest levels of expression in the aerobic and lowest levels of expression in the anaerobic conditions (cluster 16). The fold differences were 2-15 between the anaerobic and intermediate oxygen and 16 to 40-fold between the anaerobic and fully aerobic conditions. In addition, *SOL3 *was slightly (1.5-fold) upregulated in the 2.8% oxygen and fully aerobic conditions compared to lower oxygen levels. Of these genes, the expression of *GND2, TKL2 *and ORF *YGR043C *had also been measured with the TRAC method and the correlation between the Affymetrix and TRAC measurements was > 0.7.

*ZWF1 *was also measured with both Affymetrix and TRAC. With both methods *ZWF1 *expression was shown to increase 1.3-fold, compared to expression in fully aerobic cells, however, this increase was seen in cells provided with 0, 0.5 and 1.0% oxygen in the Affymetrix analysis, but only in cells provided with 2.8% oxygen in the TRAC analysis. Of the other genes from the pentose phosphate pathway, *GND1*, *TKL1 *and *TAL1 *did not show significant differences in their expression levels in different oxygen conditions when measured with Affymetrix.

### Effect of oxygen on the proteome and enzyme activities, correlated with transcriptome changes

2D-gel analysis of 2-4 independent cultures from each level of oxygen provision resulted in a proteome of 484 protein spots in total that were included in the statistical analysis. After quantile normalisation, a similar analysis for statistically significant changes in quantity with linear modelling was performed as with the gene expression data. This analysis revealed 145 spots that differed significantly (p < 0.01) when the cells were provided different levels of oxygen. Of the 484 spots, 209 were identified. The data is presented in additional data file 2.

Enzymes of the TCA cycle and those involved in respiration showed either a slight increase in quantity (1.5 to 2-fold) in the intermediate oxygen conditions, compared to other conditions (Idh2p, Mdh2p, Sdh1p, Atp3p, Atp5, Atp7p, Qcr2p, Rip1), a strong increase (3 to 64-fold) in fully aerobic conditions (Cit1p, Fum1p, Lsc1p, Idp2, Atp1, Cyb2p) or did not differ in different levels of oxygen provision (Aco1p, Idh2p, Atp2, Atp7p, Idp1p, Lsc2p). Many of the proteins involved in glucose fermentation were found as multiple pI isoforms which differed in relative quantities in different oxygen levels. These included Adh1p (3 pI isoforms), Adh2p (3), Ald4p (2), Ald6p (2), Eno1p (6), Eno2p (4), Gpm1p (3), Fba1p (2) and Hxk1 (2).

**Figure 4:**
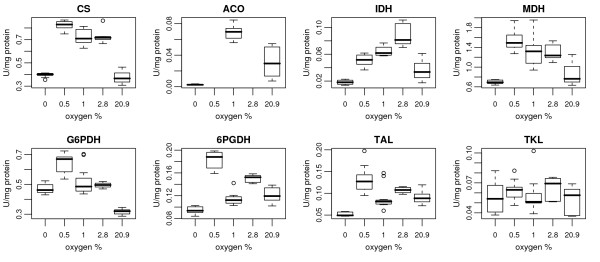
**Enzyme activity levels in 0, 0.5, 1.0, 2.8 and 20.9% oxygen**. Activity of TCA cycle enzymes citrate synthase (CS), aconitase (ACO), isocitrate dehydrogenase (IDH), malate dehydrogenase (MDH) and of the PPP enzymes glucose-6- phosphate dehydrogenase (G6PDH), 6-phosphogluconate dehydrogenase (6PGDH), transketolase (TKL) and transaldolase (TAL). The data was obtained from 2 to 4 samples taken during steady states in 2 to 4 parallel cultivations. In the boxplots the box corresponds to the IQR (inter-quartile range) and the midpoint corresponds to the sample median. The whiskers extend to extreme values of the data (within 1.5 times the IQR from the upper or lower quartile). Open circles correspond to outliers.

Enzyme activities were measured from crude cell extracts, providing a measure of the combined activity of all isoforms of the respective enzymes in the cell (Figure 4). The activities were expressed as units (U) per total soluble protein. It has previously been shown that there are only small differences in the protein content of the cells grown in aerobic and anaerobic glucose-limited chemostats at the growth rate of 0.1 h^-1.^[27]. In comparison of enzyme activities we assumed that the protein content of cells grown in oxygen limited conditions would be similar to those of cells grown anaerobically and aerobically. The activities of citrate synthase (CS), aconitase (ACO), isocitrate dehydrogenase (IDH) and malate dehydrogenase (MDH), from the TCA cycle, strongly correlated (correlation > 0.89) with the transcriptome data for the corresponding genes of the TCA cycle (*CIT1, ACO1, IDH1,2 *and *MDH1*, respectively). Of the enzymes of the pentose phosphate pathway, the activity of glucose-6-phosphate dehydrogenase (G6PDH) had a correlation of 0.7 with the corresponding gene, *ZWF1*. The activities of 6-phosphogluconate dehydrogenase (6PGDH), transketolase (TKL) and transaldolase (TAL) had a correlation of 0.5 to *GND1, TKL1 *and *TAL1*, respectively, and no correlation to *GND2, TKL2 *and ORF *YGR043C*, respectively.

**Figure 5:**
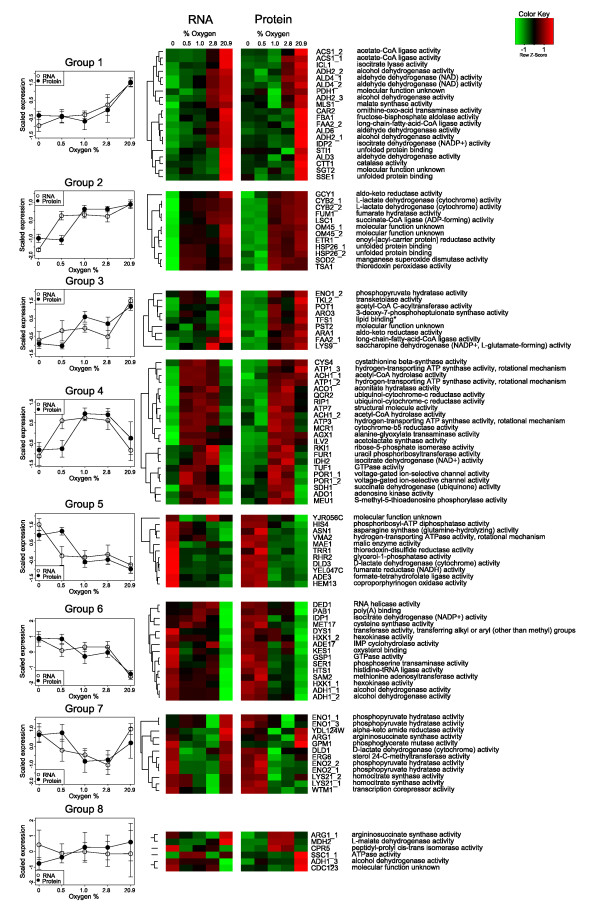
**Comparison of protein and transcript level data**. Clustering of protein spots which differed significantly in the cultures receiving different oxygen levels with their corresponding gene expression profiles. The expression values are centred and scaled around a mean of zero and standard deviation of 1. In heatmaps, green indicates low expression and red indicates high expression. For each group, the mean profile of the gene and protein expression is plotted with corresponding standard deviations. Different pI forms of the proteins are differentiated with numbers 1, 2 and 3.

In all the aeration conditions studied, the Pearson's correlation between proteins identified in the 2D gels and the mRNA levels of the corresponding genes in the transcriptome was similar, with an r-value between 0.41 and 0.55. For a more detailed comparison, the 107 significantly changing protein spots (from the 2D-gels) and the corresponding transcripts were hierarchically clustered (Figure 5). In the case of multiple protein isoforms, the corresponding transcript was assigned to each isoform separately. Of the eight groups formed by the cluster analysis, the protein and transcript quantities in groups 1 and 6 showed a high correlation (average 0.80 and 0.77, respectively). Members of group 1, related to metabolism of ethanol (ADH2), the glyoxylate cycle (ICL1, MLS1), fatty acid metabolism (FAA2), acetyl CoA synthesis (ACS1, ALD6, ALD4), and glycolysis (FBA1), were at high levels in fully aerobic conditions and both the expression of the genes and the quantity of the proteins decreased with decreasing oxygen availability. Members of group 6, involved in translation (DED1, PAB1, DYS1, HTS1) and amino acid metabolism (MET17, SER1, SAM2), glycolysis and ethanol fermentation (HXK1, ADH1), were at high levels in anaerobic conditions and on low levels in fully aerobic conditions. In groups 2, 4 and 5 the transcript and protein levels differed significantly only in cells provided with 0.5% oxygen. Group 2 contained genes and proteins involved in oxidative stress (SOD2, TSA1), redox balance (GCY1, CYB2), fatty acid metabolism (ETR1) and the TCA cycle (FUM1, LSC1). The protein levels in group 2 were high with 1.0 to 20.9% provided oxygen, while the transcript levels were already high with 0.5% provided oxygen. In group 4, related to the TCA cycle (ACO1, IDH2, SDH1), oxidative phosphorylation (ATP1, QCR2, RIP1, ATP7, ATP3) and other mitochondrial reactions (ILV2, MCR1, TUF1, POR1), the protein levels were highest with 1.0 and 2.8% provided oxygen and the transcript levels were again high already with 0.5% provided oxygen. In group 5, containing genes and proteins related to redox balancing (TRR1, RHR2, DLD3, YEL047C), the highest protein levels were observed in anaerobic conditions and when 0.5% oxygen was provided, while gene expression levels were highest under anaerobic conditions. Members of group 3, involved in various different functions, had their highest protein and gene expression levels in fully aerobic conditions, but in oxygen-restricted conditions the levels did not correlate. Group 7 contained genes and proteins, the expression and quantity of which correlated in some levels of provided oxygen. Group 8 contained genes and proteins that did not show any correlation.

## Discussion

Our results demonstrate that the oxygen limitation, not only the presence or absence of oxygen, strongly affects both the transcriptome and proteome of the yeast *S. cerevisiae*. Genes related to the respiratory pathway, the TCA cycle, metal ion homeostasis and the MAPK signalling pathways of mating and filamentous growth responded specifically to intermediate oxygen availability, a response not seen when focusing only on anaerobic and aerobic growth conditions. In addition, comparison of array and proteome data indicated post-transcriptional regulation, especially with 0.5% oxygen in the feed gas.

Respiratory functions inevitably have the highest oxygen-demand of cellular reactions. Interestingly, analysis of the transcriptome revealed an upregulation of nearly all genes encoding subunits of respiratory complexes and the main enzymes of the TCA cycle in conditions of intermediate oxygen. The differences at the transcriptional level were less than 2-fold and would have been neglected in clustering analyses involving a pre-selection of genes. The same trend was observed in the proteome as an increase in the concentration of some of the proteins of the TCA cycle and respiratory chain, and further confirmed by increased enzyme activities of the enzymes of the TCA cycle in the intermediate oxygen levels. This may indicate that the cell senses that oxygen is restricted and tries to enhance respiration by global upregulation of the genes related to these functions. Supporting this hypothesis, several genes encoding enzymes that function in the transport of iron and zinc also had their highest expression in the intermediate oxygen conditions. Proteins functioning in oxygen binding and oxygen-dependent metabolism contain a large proportion of the cellular iron, copper and other metals [37] and additional metals are thus needed to enhance the synthesis of oxygen binding proteins. To our knowledge, the global upregulation of respiratory pathways observed in this study as a response to intermediate oxygen availability has not previously been described.

The Hap2/3/4/5p transcription factor, the binding site of which was enriched among the promoters of the respiratory genes which were upregulated in the intermediate oxygen conditions, is known to act as an activator of many genes encoding subunits of the respiratory chain complexes. It may have a role in gene regulation induced by oxygen restriction, possibly in combination with regulation mediated by the two 3'UTR motifs found to be enriched in the genes that had their highest expression level in the intermediate oxygen conditions. One of these motifs (WHATATTC) resembles the motif AATATTCTT found in a comparative genomics study of three yeast species [38] and a similar motif found by the matrixREDUCE algorithm [39]. Both of these studies identified the motif as over-represented among genes related to energy metabolism, the latter being particularly over-represented among the genes of the electron transport chain.

Hap2/3/4/5p is suggested to play a role in the activation of respiration during growth rates above 0.08 h^-1 ^providing excess respiratory capacity that allows for respiratory metabolism at higher glucose fluxes [10,11]. Both high specific growth rates and intermediate oxygen provision in glucose-limited chemostats lead to onset of respirofermentative metabolism [29,40]. In fully aerobic conditions at growth rates below 0.3 h^-1^, the cell is able to maintain fully respiratory metabolism. In conditions with only 0.5 to 2.8% oxygen provided, however, the restricted oxygen provided is not sufficient for purely respiratory metabolism even though the cell responds by upregulation of Hap2/3/4/5p controlled networks.

Hap2/3/4/5p has also been suggested to control other mitochondrial processes than respiration [10] to coordinate functions of both nucleus- and mitochondrion-encoded mitochondrial proteins. However, at the transcriptional level, our data indicates that mitochondrial translation and import machineries are not co-ordinately regulated with respiratory functions, as the genes encoding the former were more highly transcribed in anaerobic rather than in fully aerobic conditions while the genes of the latter were not. In addition, the 3'UTR sites found in these groups were different. The results suggest that mitochondrial import and translational machineries have an important role also during anaerobic conditions, in which mitochondria are known to exist in the form of pro-mitochondria, the role of which is not yet very well understood [41].

Utilisation of fatty acids is an oxygen-dependent process [42]. Accordingly, the genes encoding activities of fatty acid β-oxidation and peroxisomal biogenesis were more highly expressed in the fully aerobic compared to oxygen-limited conditions. Interestingly, there was a clear difference between cells grown with 20.9% and 2.8% oxygen in the feed gas, even though the oxygen uptake rate under these two conditions was very similar. *CTA1 *and *POX1 *have previously been shown to be regulated by oxygen [12,43], via a heme-dependent pathway that does not involve Hap1p, and via another mechanism that does not rely on heme, but the exact pathways are not known [43]. It is possible that the other genes of the fatty acid β-oxidation pathway in the same cluster (*TES1, CRC1, ANT1 *and *FOX2*) are regulated by oxygen via the same, still unknown pathway as *CTA1 *and *POX1*.

Sterol biosynthesis, which is also an oxygen-requiring process, is regulated differently than fatty acid oxidation. In contrast to fatty acid oxidation, which is mainly related to energy production, sterol biosynthesis is essential for the cell [15,44,45]. Our promoter analysis revealed two putative transcription factor binding site motifs that are possibly involved in sterol metabolism. One of these (BTAWACGA) was found in 18 out of 20 promoters of the genes of ergosterol biosynthesis, in all promoters of DAN/TIR genes encoding cell wall mannoproteins, and interestingly also in *UPC2 *and *ECM22*, genes which encode transcription factors known to be involved in the regulation of sterol uptake and biosynthesis [20,46-48]. A closer look at this motif revealed that it corresponds to the AR1 (TCGTATA) and SRE (TCGTTYAG) motifs which are involved in Upc2p/Ecm22p mediated transcriptional regulation of anaerobically induced genes and *ERG *genes, respectively [20,49]. The other motif found by the FIRE programme, RACAATAG, has previously been observed through phylogenetic footprinting analysis to be enriched in genes of lipid metabolism [50] and in genes which where upregulated on galactose medium in anaerobic conditions [13]. The motif was found in the promoters of 6 *ERG *genes, *UPC2 *and *ECM22*. Thus this motif may be involved in regulation of genes of ergosterol biosynthesis directly or through Upc2p and Emc22p.

Even though the ergosterol biosynthesis pathway is not active in the absence of oxygen [51-53], some of the genes of this pathway (*ERG *genes) had higher expression in anaerobic cultures compared to the cultures receiving oxygen. This has previously been observed in cultures which were anaerobic or severely oxygen-restricted [13,21,27], but the opposite has also been reported. It was recently shown that some of the *ERG *genes are repressed by Hap1p in severely oxygen restricted conditions [8]. Anaerobic upregulation of *ERG *genes may reflect the fact that sterols are essential, by maintaining high levels of transcription for pathways of sterol and unsaturated fatty acid synthesis, priority may be given to these pathways in conditions where oxygen becomes available after a period of anaerobicity to facilitate mitochondrial membrane biogenesis [54].

Gene set enrichment analysis of our data indicated that the genes of GO category of stress response were affected by oxygen provision. The GO category of stress response contains a wide set of genes responding to different stress conditions and the cluster analysis showed that these genes were indeed enriched in three clusters with distinct expression profiles. However, all these clusters showed a higher expression level of stress-related genes in the fully aerobic conditions, compared to 2.8% provided oxygen. *XBP1*, encoding a stress-induced transcriptional repressor, was found to be regulated in an oxygen-dependent manner: the level of transcripts of this gene was lowest in anaerobic, highest in the fully aerobic and on a similar, intermediate level in the intermediate oxygen conditions. The DNA-binding domain of Xbp1 is homologous to the DNA-binding domains of cell cycle regulators Swi4p and Mbp1 and binds a related sequence [55,56]. Xbp1p has been indicated to act as a sporulation specific regulator in diploid cells, but the role of this transcription factor in haploid cells is not clear [55]. The transcription of *XBP1 *is induced by various stress situations, including glucose starvation and oxidative stress [55,56]. The enrichment of its binding sites in the promoters of the genes whose transcription negatively correlated to the transcription of this repressor, indicated a role related to cell division and cell wall organisation. However, it is not clear why these functions would be regulated in conditions of constant growth rate.

Interestingly, several genes of the MAPK signalling pathway of mating and filamentous growth were considerably upregulated in the conditions of intermediate oxygen availability. Glucose limitation is known to provoke filamentous growth in haploid cells with concomitant action of MAPK, 5'-AMP-dependent and 5'-cyclic AMP-dependent kinase pathways (reviewed in [57,58]). The MAPK signalling pathway of mating and filamentous growth may be activated by low glycosylation of the exo-cellular domain of Msb2p in low glucose conditions. However, since the glucose was below detection limit in all cultures studied, oxygen limitation in addition to glucose limitation, contributed to the induction of this signalling pathway in the conditions of intermediate oxygen availability. No clear filamentation of the cells was observed under these conditions, possibly due to lack of activity of the other kinase pathways.

In conditions of restricted respiration, the carbon flux through glycolysis is increased while the flux through the pentose phosphate pathway (PPP) remains constant [30,59-62]. The regulation of glycolysis has been shown to be predominantly post-transcriptional [27,29,63], but less is known about the regulation of the PPP. Expression of the genes encoding the main isoenzymes of the PPP did not differ significantly in conditions of different oxygen provision, while the expression of the genes encoding the minor isoenzymes of PPP, *GND2, TKL2, SOL4 *and ORF YGR043C (*NQM1) *was strongly affected by oxygen provision. The expression of the latter genes was not dependent on the absolute level of oxygen in the feed gas, but rather on the absence, limited provision or excess oxygen. Genes encoding these isoenzymes are also induced after the diauxic shift [64]. It thus seems that they are important for respiratory metabolism and that the downregulation of their expression in fermentative conditions is not dependent only on glucose repression.

We previously published transcription data for 72 selected genes related to central carbon metabolism, measured with the TRAC method [29]. Comparison of the results of TRAC analysis and the present data revealed good correlation for most of the transcripts showing more than 2-fold difference in their expression when measured with Affymetrix. This is similar to the correlation observed in comparison of transcription data for 1375 human genes using microarray or real-time PCR analysis [65]. The proportion of genes that showed similar significant differences with both arrays and real-time PCR was dramatically decreased when changes less than 2-fold were considered [65]. The differences between the TRAC and the Affymetrix measurements may be due to differences in sample preparation and the need for cDNA synthesis: in TRAC crude cell lysates are used and no cDNA synthesis is performed while in Affymetrix purified RNA is used to synthesise cDNA for hybridisation [66]. In addition, normalisation and probe design may affect the results [67].

Our results on the correlation of the proteome and transcriptome (Pearson correlation 0.41 to 0.55) are consistent with previous studies in yeast [68], although both higher [69] and lower [70] correlation has been reported. The 35 proteins spots and the corresponding genes of oxygen dependent reactions and respiration, and of acetyl-CoA synthesis, amino acid metabolism, translation and glycolysis in groups 1 and 6 were very similarly affected by the oxygen availability, suggesting regulation at the transcriptional level. However, the glycolytic enzymes in particular, as well as aldehyde dehydrogenases, were found as multiple pI isoforms and the responses of the isoforms of glycolytic enzymes differed from each other, indicating regulation by phosphorylation [71].

Interestingly, for several transcript-protein pairs the expression correlated when oxygen availability was high, but varied in low oxygen conditions, especially when only 0.5% oxygen was provided (members of groups 2, 4 and 5). Proteins of the TCA cycle and the electron transport chain, and those related to oxidative stress and additional mitochondrial functions, which clustered in groups 2 and 4, were present at relatively low levels when 0.5% oxygen was provided, although the gene expression levels were already high, suggesting post-transcriptional regulation of these proteins. In group 5, related to redox status of the cell, the opposite was observed, i.e. protein levels were high, even though gene expression was down-regulated, again indicating a post-transcriptional level of regulation. Thus, both physiological characteristics and the fluxes [29] demonstrate that provision of 0.5, 1.0 or 2.8% oxygen in the feed gas results in distinct modes of respiro-fermentative metabolism which are clearly not achieved through transcriptional regulation alone.

## Conclusion

The level of oxygen provision affected a significant part of the transcriptome of *S. cerevisiae*. However, there were only a few genes, the expression of which strictly correlated with oxygen concentration in the feed gas. Rather, the differences were observed in comparison of anaerobic, oxygen-limited and fully aerobic conditions, which require different modes of metabolism in *S. cerevisiae*. An overview of the interactions of the genes and transcription factors highlighted in this study is given in the Additional file 3 in which the relative gene expression in the different conditions is indicated along with the relevant transcription factors. Further, comparison of transcriptome and proteome level data indicated post-transcriptional regulation, especially when 0.5% oxygen was provided.

In the oxygen-limited conditions, the genes encoding respiratory pathways were more highly expressed and the quantities of proteins were higher than in either anaerobic or aerobic conditions. Regulation was possibly achieved at the transcriptional level through the action of the Hap2/3/4/5p transcription factor and the two previously undescribed 3'UTR elements. While the expression levels of these genes were high in all three oxygen-limited conditions studied, the protein quantities were high only when 1.0 or 2.8% oxygen was provided, suggesting a post-transcriptional level of regulation. In addition, according to these results, the transcriptional responses of respiratory and mitochondrial translational machineries were not coordinated, since the transcription of the latter was at a higher level in oxygen-limited and anaerobic conditions, compared to fully aerobic conditions, whereas the transcription of the former was higher in oxygen-limited conditions, compared to either anaerobic or fully aerobic conditions. Further, the regulatory elements enriched in the genes of mitochondrial translation machinery were different to those enriched in genes related to respiratory pathways.

## Methods

### Strain and culture conditions

The cultivations were described and the fermentation data published by Wiebe and co-workers [29]. Briefly, *Saccharomyces cerevisiae *CEN.PK113-1A (*MATα*, *URA3*, *HIS3*, *LEU2*, *TRP1*, *MAL2-8c*, *SUC2) *was grown in 0.8 to 1 L medium in B. Braun Biotech International (Sartorius) Biostat^® ^CT (2.5 L working volume) bioreactors in the defined minimal medium described by Verduyn *et al*. [72], with 10 g glucose l^-1 ^as carbon source, and supplemented with 10 mg ergosterol l^-1 ^and 420 mg Tween 80 l^-1^. Silicone antifoam (BDH 331512K, VWR International, UK; 0.5 mL l^-1^) was used to prevent foam production in the cultures. Chemostat cultures were maintained at D = 0.10 ± 0.02 h^-1^, pH 5.0, 30°C, with 1.5 volume gas [volume culture]^-1 ^min^-1 ^(vvm). For cultures which received less than 20.9% O_2 _(vol/vol) in the gas stream, air was replaced with the equivalent volume of N_2_, so that total gas flow was maintained constant for all experiments. Cultures which were fed 2.8 or 20.9% O_2 _were subject to oscillations. To prevent these, approximately 5% of the total cell concentration in the bioreactor was added to the culture as cells in mid to late exponential phase at the time when continuous medium feed was started [73].

### Transcriptome analysis

Affymetrix microarray analysis of two (0.5%, 2.8% O_2_) or four (0%, 1.0%, 20.9% O_2_) parallel cultivations was performed. From cultures which received 0.5% and 2.8% O_2_, two separate steady state samples were also analysed. In addition, from one of the cultivations with 1.0% O_2 _in the feed gas, four separate steady state samples were analysed. The cells were collected in cold (+4°C) 10 mM Na-phosphate buffer, pH 7. After centrifugation (3500 rpm, 5 min, +4°C), the cell pellet was frozen in liquid nitrogen. For anaerobic samples, the buffer was saturated with nitrogen in advance. For RNA extraction, 5-20 mg dry mass of cells were suspended in 400 μl cold (+4°C) disruption buffer (20 mM Tris-HCl, pH 7.4, 100 mM KCl, 2 mM MgCl_2_, 2 mM DTT). 400 μl phenol-chlorophorm (50:50), 5 μl 20% (w/v) SDS and 400 μl glass beads (0.5 mm diameter, Biospec Products) were added. The cells were disrupted with a Fastprep machine (Q-Biogene), 2 × 20 s, at speed 6. After centrifugation (14000 rpm, 15 min, +4°C), supernatant was used for total RNA extraction. The total RNA extraction was done with an RNeasy kit (Qiagen) according to manufacturer's instructions.

Hybridisations were carried out at the Finnish DNA Microarray Centre at Turku Centre for Biotechnology. 2 μg of total RNA was used as starting material for sample preparation. Samples were processed according to the One-Cycle Target Labelling protocol in the GeneChip Expression Analysis Manual (Affymetrix). Both before and after the amplifications the total RNA/cRNA concentrations were assessed with Nanodrop ND-1000 and total RNA/cRNA quality was assessed by BioRad's Experion electrophoresis station. Each sample was hybridised to the GeneChip Yeast Genome 2.0 Array at +45°C overnight (16 h) according to the GeneChip Expression Analysis Technical Manual (Affymetrix). A GeneChip Fluidics Station 450 was used to wash and stain the arrays, and a GeneChip Scanner 3000 with AutoLoader was used to scan the arrays. CEL-files were extracted with GCOS Manager 1.4.

All data analysis was done using R/Bioconductor, version 2.5.1 [74,75]. The raw data was normalised with Robust Multichip Average (RMA) normalisation [76]. Statistical differences in the expression were analysed using linear modelling with the tools of limma package [77]. For each gene, a linear model was fitted by the least squares method and differential expression within pairs of experimental conditions was computed using the empirical Bayesian approach [78]. For correction of multiple testing errors, the Benjamini & Hochberg -method controlling false discovery rate (FDR) was used [79]. The microarray data can be accessed through GEO accession number GSE12442.

The Gene Ontology (GO) classes and KEGG pathways differentiating the conditions studied were computed using parametric gene set enrichment analysis [33,80]. Pair wise fold changes between conditions of different oxygen provision were calculated and the fold changes were used to calculate Z scores for the gene sets. Statistical significances of Z scores were determined against normal distribution.

The clustering analysis of gene expression data was performed using fuzzy c-means clustering [34,81]. The clustering method assigns genes to clusters with gradual membership (values between 0 and 1). For the clustering, the expression values were scaled and centred to have a mean of zero and standard deviation of one. The parameter m, which controls the sensitivity of the clustering process to noise, was adjusted to 1.25 to prevent the detection of clusters in randomised data. The number of clusters was selected, such that no clusters were formed where all the genes would have membership values below 0.5. The enriched GO classes and KEGG metabolic pathways in the clusters were computed with the GOstats package [82]. Transcriptional regulatory motifs in the clusters were analysed with the FIRE method [35].

### Proteome analysis

Cells for proteome analysis were collected in cold Na-phosphate buffer, pH 7 as described for the microarray analysis. 5-10 mg dry mass of cells was re-suspended in 150 μl of 10% (v/v) trichloro acetic acid (TCA, Merck) in 1.5 ml micro centrifuge tubes. 500 μl glass beads (0.5 mm diameter, Biospec Products) were added and the tube inserted into a MiniBeadbeater 8 (Biospec Products) and shaken at homogenisation speed, three times for 30 seconds. The tubes were cooled on ice between each homogenisation step. The suspensions were withdrawn and proteins were precipitated by adding 600 μl of -20°C acetone and incubating 30 min. on ice. Precipitated proteins were collected by centrifugation for 30 min., 13 000 rpm, at 4°C, rinsed once with 600 μl of -20°C acetone and re-suspended in 450 μl of 7 M urea (Promega, USA), 2 M thiourea (Fluka, USA), 4% (w/v) CHAPS (Fluka), 1% (w/v) Pharmalytes 3-10 (Pharmacia, Sweden) and 1% (w/v) DTT (Sigma) by gently shaking for 20 min. at room temperature. Supernatants were collected by centrifugation for 5 min. 13 000 rpm (Eppendorf bench centrifuge). The protein concentration of supernatants was determined by the Non-Interfering Protein Assay (Geno Technology, Inc.) and the samples were stored at -70°C before isoelectric focusing.

Isoelectric focusing and second dimension 11% (w/v) SDS-PAGE were carried out as described earlier [83]. After electrophoresis the gels were fixed for one and a half hours in 30% (v/v) ethanol and 0.5% (v/v) acetic acid and stained with Sypro Ruby (Molecular Probes), according to manufacturers' instructions. The stained gels were scanned with a resolution of 100 microns on a Typhoon instrument (GE Healthcare). The gel images were analysed using the Progenesis software (Nonlinear Dynamics). The gel patterns from different gels were automatically matched, with some additional manual editing, and the quantities of matching spots in different gels were compared. From each condition, samples from 2-4 independent cultivations were used and for each sample 4 gels were analysed. After background correction, the data was transferred to the R environment for normalisation and data analysis.

Background corrected proteome data of 500 spots was obtained from Progenesis software. For each condition, the spots that had zero values in more than 50% of the gels were treated as real zeroes and set to the lowest value of each gel [84]. The rest of the missing values were estimated using the k- nearest neighbour-method [85]. The data was log transformed and quantile normalised as described in [86]. The statistical analysis of differences was done using linear modelling [77].

Protein identifications were carried out in the Protein Chemistry Unit, Institute of Biomedicine, Anatomy, Biomedicum, Helsinki. For protein identification, excised gel spots were washed and dehydrated with acetonitrile (Rathburn, Scotland, HPLC grade S). Proteins were reduced with 20 mM DTT and incubated at 56°C for 30 min before alkylation with 55 mM Iodoacetamide (Sigma, USA)/100 mM ammonium hydrogen carbonate (NH_4_HCO_3_) in the dark at room temperature for 15 min. After washing with 100 mM NH_4_HCO_3 _and dehydration with acetonitrile the gel pieces were rehydrated in 10 to 15 μl sequencing grade trypsin (Promega, USA) in 100 mM NH_4_HCO_3_, to a final concentration of 0.01 μg/μl trypsin and incubated for trypsin digestion overnight at 37°C. Tryptic peptides were eluted from the gel pieces by incubating for 15 min at room temperature successively in 25 mM NH_4_HCO_3 _and then twice in 5% formic acid. The tryptic peptides were desalted using Zip Tip μC-18 reverse phase (Millipore, USA) and directly eluted with 50% v/v acetonitrile/0.1% v/v trifluoroacetic acid (TFA) onto a MALDI target plate. Then, a saturated matrix solution α-cyano-4-hydroxy cinnamic acid (CHCA) (Sigma, USA) in 33% ACN/0.1% TFA was added. MALDI-TOF analyses were carried out with an Autoflex (Bruker Daltonics, Bremen Germany) equipped with a nitrogen pulsed laser (337 nm) and operating in positive mode. Typically, mass spectra were acquired by accumulating spectra of 240 laser shots. External calibration was performed for molecular assignments using a peptide calibration standard (Bruker Daltonics GmbH, Leipzig, Germany). Trypsin autolytic peptide masses were used to check or correct the calibration.

Protein identifications were performed by searching the peptide masses against the National Center for Biotechnology Information (NCBI) non-redundant database using Matrix Science's Mascot - Peptide Mass Fingerprint . Protein identifications by peptide mass fingerprinting were further evaluated by comparing the calculated and observed molecular mass and pI, as well as the number of peptides matched and percent sequence coverage.

Comparison of proteome and transcriptome data was done according to Gallardo and co-workers [87]. Significantly (p < 0.01) changing protein spots (107) and the corresponding genes were clustered using a hierarchical clustering with average linkage method and correlation as distance metric. Visualisation was done in R/Bioconductor and Inkscape version 0.46 [88].

### Enzyme activity assays

Enzyme activities were measured as units (U) per mg of total soluble protein. One U was defined as the activity which converts one μmol substrate per min. Enzyme activities were measured from cell extracts prepared by disrupting the yeast cells with glass beads in 100 mM Hepes-buffer, pH 7.6, 1 mM DTT, supplemented with Complete protease inhibitor cocktail (Roche Applied Science, USA). The protein concentration of the cell extracts was determined with the Bio-Rad Protein Assay (Bio-Rad Laboratories, USA), using bovine serum albumin as the standard. All enzyme activities were measured as triplicates using the Konelab Arena 20XT automated analyser (Thermo Scientific, Finland) at 30°C and 340 nm in 79 mM TEA, pH 7.6, except citrate synthase activity which was measured at 420 nm. The isocitrate dehydrogenase (EC 1.1.1.41) assay mixture contained 1.2 mM MnCl_2 _and 0.48 mM NAD. The reaction was started by adding DL-isocitrate trisodium salt to a final concentration of 4 mM. The citrate synthase (EC 4.1.3.7) assay mixture contained 0.38 mM Acetyl-CoA and 0.1 mM DTNB. The reaction was started by addition of oxaloacetic acid to a final concentration of 0.5 mM. The aconitase (EC 4.2.1.3) assay mixture contained 1.2 mM MnCl_2_, 0.48 mM NADP and 0.8 U ml^-1 ^isocitrate dehydrogenase (NADP dependent). The reaction was started by addition of sodium citrate to a final concentration of 2 mM. The malate dehydrogenase (EC 1.1.1.37) assay mixture contained 0.2 mM NADH. The reaction was started by addition of oxaloacetic acid to a final concentration of 0.56 mM. The transketolase (EC 2.2.1.1) and transaldolase (EC 2.2.1.2) assay mixture contained 0.2 mM NADH, 20 U ml^-1 ^triosephosphate isomerase and 20 U ml^-1 ^glycerolphosphate isomerase. In addition, transketolase mixtures contained 0.3 mM thiamine pyrophosphate and 4 mM MgCl_2_. The transketolase assay was started by addition of ribulose-5P and ribose-5P to final concentrations of 5 mM and ribulose epimerase to 25 U ml^-1^. The transaldolase assay was started by addition of erythrose-4 phosphate and fructose-6 phosphate to final concentrations of 0.7 mM and 5.4 mM, respectively. Glucose-6 phosphate dehydrogenase and 6-phosphogluconate dehydrogenase assay mixtures contained 0.2 mM NADP and 8 mM MgCl_2_. The reactions were started by addition of glucose-6 phosphate and 6-phoshphogluconate, respectively, to final concentrations of 0.3 mg ml^-1^.

## Authors' contributions

ER, MT, LR and MP conceived the study. ER and MT carried out the transcriptome and proteome analyses and drafted the manuscript. MGW, ER and MT carried out the fermentations and MGW revised the manuscript. ER performed the enzyme activity measurements. J-PP participated in the statistical analysis and preparation of Figure S1. LR supervised the work and revised the manuscript. All authors read and approved the final manuscript.

## Supplementary Material

Additional file 1 **Over-represented GO- and KEGG-classes in clusters as determined by fuzzy c-means clustering of gene expression in cells receiving 0, 0.5, 1.0, 2.8 or 20.9% oxygen, and illustrated in Figure**2. Click here for file

Additional file 2 **Total soluble protein data. Normalised spot intensities and corresponding standard deviations in cells receiving 0, 0.5, 1.0, 2.8 or 20.9% oxygen**. Click here for file

Additional file 3 **Overview of gene expression data in cells receiving 0, 0.5, 1.0, 2.8 or 20.9% oxygen of genes encoding the main metabolic pathways and oxidative phosphorylation of*****Saccharomyces cerevisiae *****and the transcription factors known to regulate these genes**. Click here for file
