# Supplementary material for: Low oxygen levels as a trigger for enhancement of respiratory metabolism in Saccharomyces cerevisiae
Source: BMC Genomics. 2009 Oct 5;10:461. doi: 10.1186/1471-2164-10-461 (PMC2767370; doi:10.1186/1471-2164-10-461)
Supplement: Additional file 1 — Over-represented GO- and KEGG-classes in clusters as determined by fuzzy c-means clustering of gene expression in cells receiving 0, 0.5, 1.0, 2.8 or 20.9% oxygen, and illustrated in Figure2. [file 1471-2164-10-461-S1.PDF]

Table S1. **Over-represented GO- and KEGG-categories in clusters of gene expression data**
Clusters were determined by fuzzy c-means clustering of gene expression in 0, 0.5, 1, 2.8 and 20.9 % oxygen, and illustrated in Figure 2.

**Cluster** (unknowns/genes)	**GO-category (p<0.01)**	**KEGG-category (p<0.01)**
**1** (10/75)	cytokinesis<br>chromatin silencing at silent mating-type cassette<br>disaccharide metabolic process	
**2** (10/186)	translation<br>biosynthetic process<br>protein metabolic process<br>ribosome biogenesis and assembly<br>telomere organization and biogenesis<br>fatty acid elongation	Ribosome
**3** (21/99)	sterol metabolic process<br>alcohol metabolic process<br>protein catabolic process<br>response to stress<br>exocytosis<br>endocytosis	Butanoate metabolism<br>Proteasome
**4** (21/163)	translation<br>protein complex assembly<br>mitochondrion organization and biogenesis<br>regulation of cyclin-dependent protein kinase activity<br>regulation of cell cycle<br>phosphatidylserine metabolic process<br>cellular protein metabolic process<br>reproductive process<br>cell wall organization and biogenesis<br>coenzyme biosynthetic process	Cell cycle<br>Glycerophospholipid metabolism
**5** (17/167)	ribosome biogenesis and assembly<br>translation<br>IMP biosynthetic process<br>amino acid and derivative metabolic process<br>carboxylic acid metabolic process<br>spermine metabolic process<br>pyrimidine transport<br>apoptosis<br>biopolymer metabolic process<br>cell organization and biogenesis	Purine metabolism<br>Aminoacyl-tRNA biosynthesis<br>Methionine metabolism<br>RNA polymerase<br>Pyrimidine metabolism
**6** (6/50)	regulation of small GTPase mediated signal transduction<br>telomere maintenance via telomerase<br>regulation of nucleic acid metabolic process<br>filamentous growth<br>regulation of amino acid metabolic process<br>fatty acid desaturation<br>transcriptional preinitiation complex formation	
**7** (50/223)	coenzyme catabolic process<br>neutral amino acid transport<br>lipid metabolic process<br>carboxylic acid transport<br>tricarboxylic acid cycle intermediate metabolic process<br>prospore formation<br>glyoxylate cycle<br>second-messenger-mediated signaling	
**8** (39/149)	trehalose metabolic process<br>protein refolding<br>response to stress<br>vacuolar protein catabolic process<br>autophagy<br>glutamate catabolic process<br>glucose metabolic process<br>regulation of gluconeogenesis<br>energy derivation by oxidation of organic compounds<br>MAPKKK cascade during cell wall biogenesis<br>alcohol biosynthetic process<br>response to abiotic stimulus<br>endocytosis	Starch and sucrose metabolism
**9** (25/86)	glycerolipid metabolic process<br>response to heat<br>response to acid	
**10** (24/209)	translation<br>macromolecule biosynthetic process<br>protein metabolic process<br>protein import into mitochondrion<br>siderophore transport<br>heme a metabolic process<br>very-long-chain fatty acid metabolic process<br>di-, tri-valent inorganic cation transport	Aminoacyl-tRNA biosynthesis<br>Selenoamino acid metabolism<br>Sulfur metabolism

Table S1. continued

Cluster (unknowns/genes)	GO-class (p<0.01)	KEGG-class (p<0.01)
**11** (30/204)	oxidative phosphorylation<br>tricarboxylic acid cycle<br>response to pheromone<br>glutamate metabolic process<br>cell division<br>signal transduction during filamentous growth<br>metal ion transport	Oxidative phosphorylation<br>MAPK signaling pathway<br>Pyruvate metabolism<br>Citrate cycle (TCA cycle)
**12** (31/113)	nonprotein amino acid metabolic process<br>ornithine metabolic process<br>monosaccharide transport<br>cell differentiation<br>transcription initiation from RNA polymerase II promoter	Urea cycle and metabolism of amino groups<br>Arginine and proline metabolism
**13** (27/267)	ribosome biogenesis and assembly	RNA polymerase<br>Pyrimidine metabolism<br>Purine metabolism<br>Aminoacyl-tRNA biosynthesis<br>Ribosome
**14** (46/189)	sterol biosynthetic process<br>lipid biosynthetic process<br>alcohol metabolic process<br>transposition, DNA-mediated<br>secretory pathway<br>biofilm formation<br>peptide transport	Biosynthesis of steroids
**15** (13/60)	DNA packaging<br>chromosome organization and biogenesis	
**16** (58/219)	fatty acid oxidation<br>peroxisome organization and biogenesis<br>sporulation<br>NADP metabolic process<br>glycogen metabolic process<br>monosaccharide metabolic process<br>pentose-phosphate shunt<br>fatty acid transport<br>response to stress	Pentose phosphate pathway<br>Glutathione metabolism
**17** (9/58)	intracellular transport<br>RNA transport<br>U2-type nuclear mRNA branch site recognition<br>protein transport<br>DNA packaging<br>nuclear translocation of MAPK<br>secretory pathway	Protein export
**18** (14/87)	mitochondrial transport<br>generation of precursor metabolites and energy<br>mitochondrial membrane organization and biogenesis<br>monovalent inorganic cation transport	
**19** (8/37)	secretory pathway<br>protein amino acid terminal glycosylation	Proteasome
**20** (27/253)	secretory pathway<br>regulation of cellular pH<br>intracellular transport<br>glycoprotein biosynthetic process	N-Glycan biosynthesis
**21** (47/232)	peroxisome organization and biogenesis<br>monocarboxylic acid metabolic process<br>propionate metabolic process<br>tricarboxylic acid cycle intermediate metabolic process<br>asparagine biosynthetic process from oxaloacetate<br>endosome transport<br>carnitine metabolic process	Glyoxylate and dicarboxylate metabolism<br>Phenylalanine metabolism<br>Tyrosine metabolism
**22** (23/145)	chromosome segregation<br>intracellular mRNA localization<br>serine family amino acid biosynthetic process<br>microtubule-based process<br>ER to Golgi vesicle-mediated transport<br>nucleotide transport<br>mitotic cell cycle<br>protein amino acid glycosylation<br>biopolymer glycosylation<br>purine base biosynthetic process	Ether lipid metabolism
