# Supplementary material for: Low oxygen levels as a trigger for enhancement of respiratory metabolism in Saccharomyces cerevisiae
Source: BMC Genomics. 2009 Oct 5;10:461. doi: 10.1186/1471-2164-10-461 (PMC2767370; doi:10.1186/1471-2164-10-461)
Supplement: Additional file 3 — Overview of gene expression data in cells receiving 0, 0.5, 1.0, 2.8 or 20.9% oxygen of genes encoding the main metabolic pathways and oxidative phosphorylation ofSaccharomyces cerevisiae and the transcription factors known to regulate these genes. [file 1471-2164-10-461-S3.PDF]

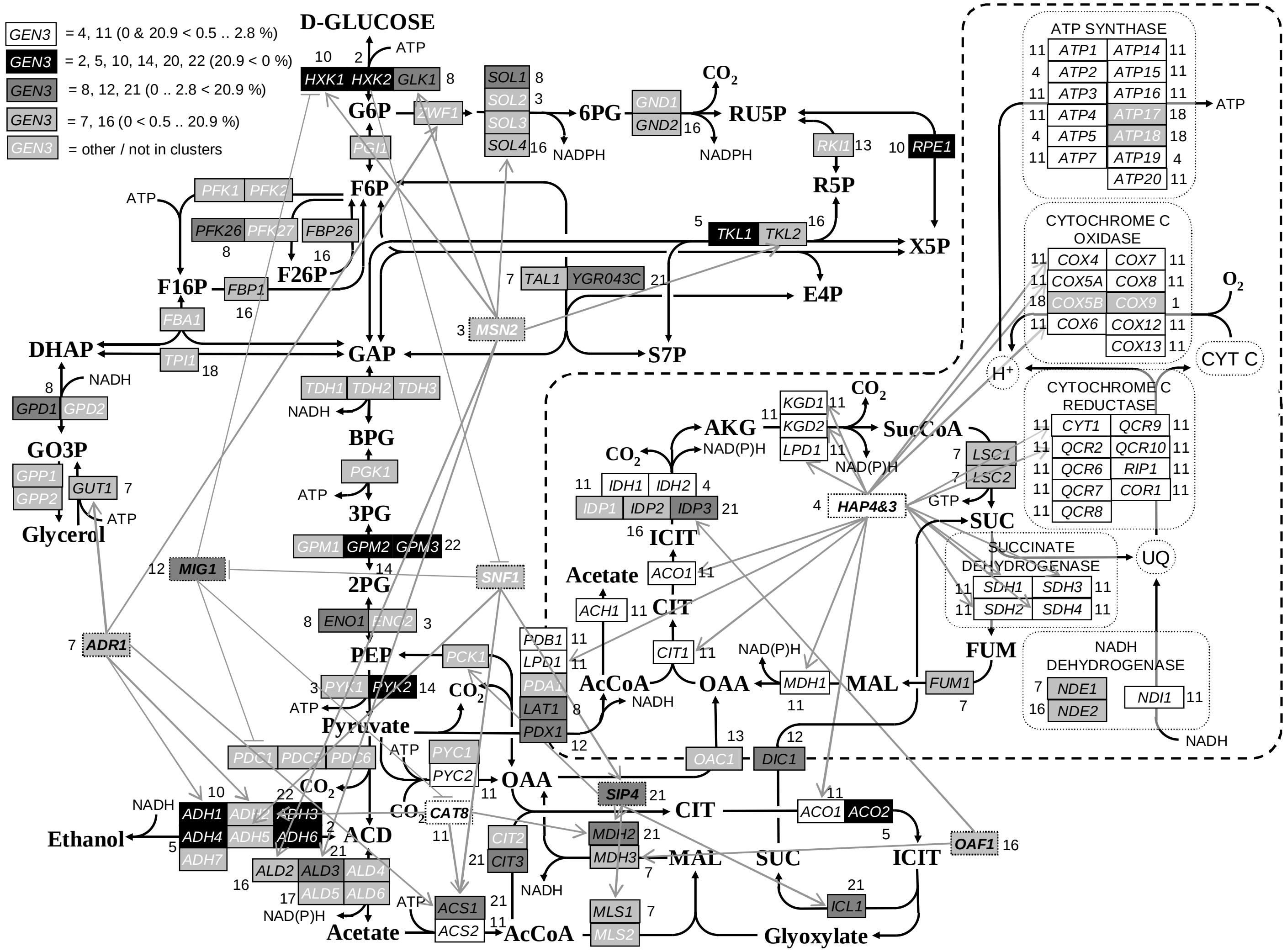

Figure S1. Main metabolic pathways and oxidative phosphorylation of *Saccharomyces cerevisiae* are presented as black lines with boxes containing the names of the genes encoding the main enzymes. Reactions and gene names were assembled from KEGG. Grey lines link transcription factors to genes, based on the Yeast Proteome Database (YPD). The expression cluster (Figure 2) the gene belongs to is indicated by the colour of the box. Numbers next to the gene boxes display the actual number of the gene cluster. White = clusters 4 and 11 in which expression in both anaerobic and aerobic conditions is less than in oxygen-limited conditions, black = clusters 2, 5, 10, 14, 20 and 22 in which expression in aerobic condition is less than in anaerobic conditions, dark grey = clusters 8, 12 and 21 in which expression is less in anaerobic and all oxygen-limited conditions than in aerobic conditions, medium grey = clusters 7 and 16 in which expression is less in anaerobic condition than in all oxygen-limited and aerobic conditions, light grey = genes in other clusters or not found in any of the 22 clusters.
